# Mouse anti-RANKL antibody delays oral wound healing and increases TRAP-positive mononuclear cells in bone marrow

**DOI:** 10.1007/s00784-015-1550-0

**Published:** 2015-08-09

**Authors:** Shinichiro Kuroshima, Zeina Al-Salihi, Junro Yamashita

**Affiliations:** Department of Applied Prosthodontics, Graduate School of Biomedical Sciences, Nagasaki University, 1-7-1, Sakamoto, Nagasaki, Nagasaki 852-8588 Japan; Department of Biologic and Materials Sciences, Prosthodontic Division, School of Dentistry, University of Michigan, 1011 North University Ave., Ann Arbor, MI 48109 USA

**Keywords:** Antiresorptives, Mouse anti-RANKL monoclonal antibody, Wound healing, Inflammation, TRAP-positive mononuclear cells

## Abstract

**Objectives:**

Denosumab, a human monoclonal antibody (mAb) that neutralizes receptor activator for nuclear factor κB ligand (RANKL), is associated with osteonecrosis of the jaw. However, the effect of denosumab on oral wounds is unclear. The aim was to determine the effect of anti-RANKL mAb on oral wounds and bone marrow.

**Materials and methods:**

The direct effect of the mAb on fibroblasts, macrophages, and osteoclasts were assessed in vitro. In vivo, mouse anti-RANKL mAb was administered to mice for 9 weeks prior to palatal bone denudation surgery. Mice were euthanized 3 weeks post-surgery, and wound healing was histomorphometrically analyzed. Long bones were assessed using micro-computed tomography, quantitative real-time polymerase chain reaction, and flow cytometry.

**Results:**

The mAb had no effect on macrophages and fibroblasts but significantly suppressed osteoclast proliferation in vitro. The mAb treatment significantly increased bone mass by suppressing osteoclasts in vivo. The expression of pro-osteoclastic genes was promoted in the bone marrow of the mAb-administered animals. Consistently, the mAb significantly induced the development of tartrate-resistant acid phosphatase (TRAP)-positive mononuclear cells (MNCs) but not osteoclasts in bone marrow. The mAb treatment had no effect on gross healing of the palatal wounds. However, significant inflammation was retained in the connective tissue facing the once denuded bone surface.

**Conclusions:**

Repair of the damaged palate was delayed, and significant inflammation was sustained in the connective tissue by anti-RANKL mAb treatment.

**Clinical relevance:**

Denosumab impairs osteoclastic bone repair. Care should be exercised to minimize osseous trauma when invasive procedures are performed on patients taking denosumab.

## Introduction

Osteoclasts are multinucleated bone-resorbing cells derived from the myeloid lineage that play critical roles in skeletal growth/remodeling, hematopoiesis, bone fracture healing, and bone diseases such as osteoporosis, Paget’s disease of bone, and multiple myeloma. It has been demonstrated that long-term suppression of osteoclasts by bisphosphonate treatment increases non-attached osteoclasts in bone marrow, i.e., cells are not directly on bone surfaces [1–3]. Similarly, we have found that long-term bisphosphonate treatment is associated with increased numbers of tartrate-resistant acid phosphatase (TRAP)-positive mononuclear cells (MNC) in mouse bone marrow [4]. It was further demonstrated that antiresorptive treatment with bisphosphonates has a negative impact on oral wound healing; bisphosphonate treatment increases inflammatory cell infiltration and reduces collagen apposition in wounds [5, 6]. Thus, osteoclast suppression by potent bisphosphonates alters the cellular environment in bone marrow as well as in oral wound healing. However, whether these findings are specifically linked to osteoclast suppression or bisphosphonates themselves is unclear. If increased TRAP(+) MNCs and altered oral wound healing are associated with osteoclast suppression, not specifically bisphosphonates themselves, then similar observations should be found when another class of potent antiresorptives, denosumab, is used.

Denosumab is a human monoclonal antibody to the receptor activator of nuclear factor-κB ligand (RANKL) that potently suppresses bone resorption by targeting osteoclasts [7]. It has been approved for the treatment of bone diseases such as osteoporosis, metastatic bone diseases, and multiple myeloma in the USA and other countries [7, 8]. Denosumab is a potent antiresorptive with a different mechanism of action from bisphosphonates. Bisphosphonates bind to bone mineral. During bone resorption, osteoclasts take up bisphosphonates together with bone mineral. Internalized bisphosphonates induce osteoclast apoptosis, thereby inhibiting further resorption [9]. Thus, bisphosphonates target active bone-resorbing osteoclasts. On the other hand, denosumab attaches to RANKL and hinders its ability to bind to its receptor, RANK, which is expressed on mature osteoclasts and their precursors [10]. Since RANKL–RANK binding is essential for osteoclast differentiation and survival, the disruption of this binding profoundly hinders osteoclast development, hence bone resorption. Thus, denosumab targets not only active bone-resorbing osteoclasts but pre-osteoclasts as well. Although the mechanism of the antiresorptive action is distinct between denosumab and bisphosphonates as described above, the literature indicates that denosumab treatment is also associated with the development of medication-related osteonecrosis of the jaw (MRONJ) which is a rare complication typically related to bisphosphonates [11, 12]. This suggests that denosumab and bisphosphonates may share a mechanism that contributes to the development of MRONJ. In this study, we hypothesized that denosumab treatment increases TRAP(+) MNCs in bone marrow and oral wounds. To test the hypothesis, mice were treated with mouse monoclonal antibody to RANKL (anti-RANKL mAb). The effects of the anti-RANKL mAb treatment on bone marrow and oral wound healing were examined.

## Materials and methods

### Animals, injections, and oral wounds

**Fig. 1:**
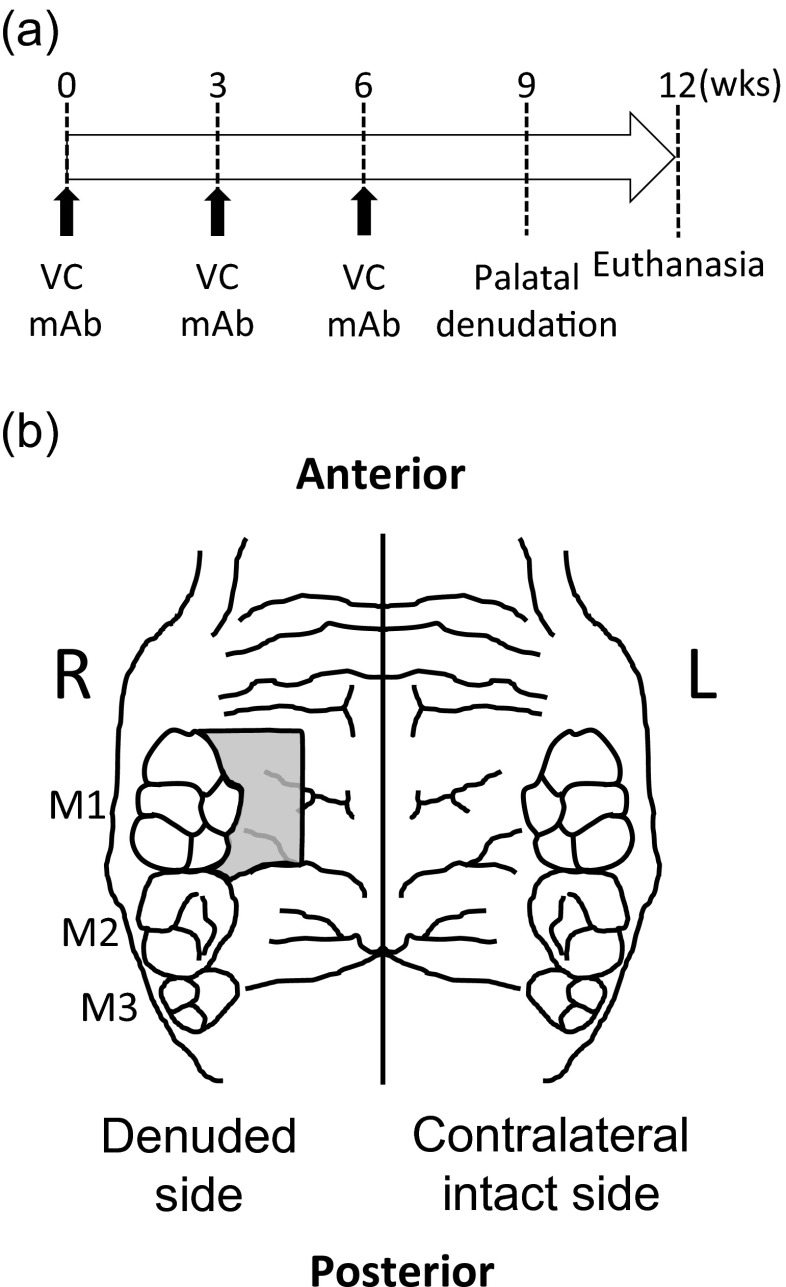
Experimental design and palatal wounds. Mouse anti-RANKL mAb (*mAb*) and saline (vehicle control, *VC*) injections were performed subcutaneously once every 3 weeks for 9 weeks. The palate was denuded at 9 weeks and left as an open wound. Mice were euthanized 3 weeks after the surgery (**a**). The denuded palate is shown. The area was surrounded by the M1, rugae, and great palatine canal (**b**)

Fourteen C57BL/6 J mice (9-week-old) were obtained from the Jackson Laboratory (Bar Harbor, ME, USA) and maintained in a temperature-controlled room with 12 h/12 h light/dark cycles. Mice were allowed to access water and standard diet ad libitum. A mouse anti-RANKL mAb (OYC Americas, Andover, MA, USA) was subcutaneously injected to half of the mice at 5 mg/kg once every 3 weeks for 9 weeks (mAb). Saline was injected to the remaining half as vehicle control (VC) (Fig. 1a). The experimental protocol was approved, and animals were treated in accordance with the guideline of the University Committee on Use and Care of Animals. Oral wounds were created at 9 weeks after the initiation of the treatment. Mice received general anesthesia (ketamine–xylazine cocktail), and the palate next to the first molar (M1) was denuded by excising a portion of the mucosa and underlying periosteum (Fig. 1b). The contralateral intact side served as an internal control. Mice were euthanized by CO_2_ asphyxiation at 3 weeks after surgery.

### Histomorphometric analysis

At sacrifice, maxillae and left tibiae were harvested, fixed in 10 % formalin, and decalcified in 10 % ethylenediaminetetraacetic acid. The decalcified maxillae and tibiae were processed for paraffin sections. TRAP staining was performed to identify osteoclasts and TRAP(+) MNCs using a commercial kit (386A, Sigma-Aldrich, St. Louis, MO, USA). TRAP-stained sections were photomicrographed, and osteoclasts on the bone surfaces, non-attached osteoclasts, and TRAP(+) MNCs within 100 μm of the bone surfaces were histomorphometrically quantified using Image-Pro Plus (Media Cyberrnetics, Bethesda, MD, USA). Masson’s trichrome staining was conducted using a commercial kit (HT15, Sigma-Aldrich, USA) to assess inflammation. Inflammatory cell infiltration was quantified in the palatal connective tissue facing towards the once exposed bone surfaces. Empty osteocyte lacunae were enumerated in the bone area exposed to the oral environment after denuding. Epithelial thickness was measured in the healing wounds. The contralateral intact side was examined for the same parameters as well.

### Microcomputed tomography

The right femur was fixed in 10 % formalin and subjected to trabecular bone analysis in microcomputed tomography (microCT) (μCT-100; Scanco Medical, Bruttisellen, Switzerland) at 10-μm voxel resolution with an energy level of 70 kV. The distal metaphysis was scanned for 1.5 mm proximal to the growth plate. Reconstructed images were segmented by the semi-manual contouring method and analyzed for the trabecular bone structure using the built-in Scanco software.

### Flow cytometry

Fresh bone marrow was harvested from the left femur, and MNCs were isolated after red blood cell lysis of the bone marrow using ACK buffer (Invitrogen). MNCs (1 × 10^6^) were incubated with phycoerythrin (PE) anti-mouse CD3e (clone: 145-2C11) and γδTCR (clone: GL3). PE Hamster IgG was used for isotype controls. Cell analysis of CD3(+) and γδTCR(+) was performed using the C6 Flow Cytometer (Accuri, Ann Arbor, MI, USA). All antibodies for flow cytometry were purchased from eBiosciences (San Diego, CA, USA).

### Serum calcium

Serum was isolated from harvested blood at euthanasia and kept at −80 °C until use. Serum calcium levels were assessed using a commercially available calcium assay kit (Pointe Scientific, Canton, MI, USA).

### In vitro assays

Bone marrow cells were isolated from the right tibiae and cultured overnight in DMEM supplemented with 10 % fetal bovine serum (FBS), 100 U/mL of penicillin, and 100 μg/mL of streptomycin. Suspension cells were then collected, plated at 2.5 × 10^5^ cells/cm^2^, and treated with 50 ng/mL of macrophage colony-stimulating factor (M-CSF; Peprotech, Rocky Hill, NJ, USA) every 3 days to induce and maintain macrophages. Macrophages were plated at 2.0 × 10^4^ cells/cm^2^ and cultured in the supplemented DMEM containing M-CSF (50 ng/mL). The anti-RANKL mAb treatment (0, 1, or 10 μg/mL) was initiated at day 0. At day 3, either cell counting or RNA extraction was performed. For osteoclast cultures, macrophages were plated at 2.0 × 10^4^ cells/cm^2^ in the supplemented DMEM with M-CSF (50 ng/mL) and RANKL (50 ng/mL) (Peprotech). Medium was replaced every 2 days. At day 5, osteoclast cultures were treated with the anti-RANKL mAb (0, 1, or 10 μg/mL) for 24 h. At day 6, either TRAP staining or RNA extraction was performed. Small pieces of gingiva were harvested from mice, and organ cultures were performed to obtain gingival fibroblasts. Fibroblasts were plated at 2.0 × 10^4^ cells/cm^2^ and cultured in supplemented DMEM containing the mouse anti-RANKL mAb (0, 1, or 10 μg/mL). Medium was replaced every 2 days. Cells were enumerated at days 2, 4, and 6. In addition, total RNA was extracted using the phenol/chloroform method. RNA was stored at −80 °C until use.

### Quantitative real-time polymerase chain reaction

Fresh bone marrow was isolated from the right tibia directly into TRIZOL (Invitrogen, Grand Island, NY, USA) using a centrifugation method [5]. Total RNA was extracted and stored at −80 °C until use. Bone marrow RNA as well as total RNA from the in vitro experiments was analyzed for gene expression. First-strand cDNA was synthesized using the SuperScript First-Strand system (Invitrogen). Quantitative real-time polymerase chain reaction (qPCR) was performed using an iCycler IQ (BioRad, Hercules, CA, USA) with SYBR green (Invitrogen). The samples were run in triplicate, and results were normalized by glyceraldehyde 3-phosphate dehydrogenase (*GAPDH*) expression. Relative quantification of data generated using this system was performed using the standard curve method. Primer sets used were *GAPDH*, *C-fms*, *RANK*, *RANKL*, *CTSK*, *Dcstamp*, *Bcl2*, *Bax*, *Bak*, *Bim*, and *Bad* (Table 1).

**Table 1:** Quantitative real-time PCR primer design

Gene	Forward	Reverse
*GAPDH*	ACCCAGAAGACTGTGGATGG	CACATTGGGGGTAGGAACAC
*C-fms*	AGGCAGGCTGGAATAATCTGACCT	CGTCACAGAACAGGACATCAGAGC
*RANK*	CAGCAGCCAAGGAGGACTAC	ACATAGCCCACACCGTTCTC
*RANKL*	CAGAAGACAGCACTCACTGC	ATGGGAACCCGATGGGATGC
*CTSK*	CAGCAGAACGGAGGCATTGA	CCTTTGCCGTGGCGTTATAC
*Dcstamp*	GGGCACCAGTATTTTCCTGA	TGGCAGGATCCAGTAAAAGG
*Bcl2*	GATCCAGGATAACGGAGGCT	GGTCTTCAGAGACAGCCAGG
*Bax*	AAACTGGTGCTCAAGGCCCT	AGCAGCCGCTCACGGAG
*Bak*	TATTAACCGGCGCTACGACAC	CTTAAATAGGCTGGAGGCGATCTT
*Bim*	CGACAGTCTCAGGAGGAACC	CCTTCTCCATACCAGACGGA
*Bad*	GTACGAACTGTGGCGACTCC	GAGCAACATTCATCAGCAGG

### Statistical analysis

All data sets were tested for equality of variances. Independent *t-*test and one-way analysis of variance (ANOVA) for parametric data and Kruskal–Wallis test for non-parametric data were performed using SYSTAT (Systat Software, Chicago, IL, USA). An *α*-level of 0.05 was used. Results are presented as mean ± SEM.

## Results

### Anti-RANKL mAb suppressed osteoclasts but not macrophages and gingival fibroblasts in vitro

**Fig. 2:**
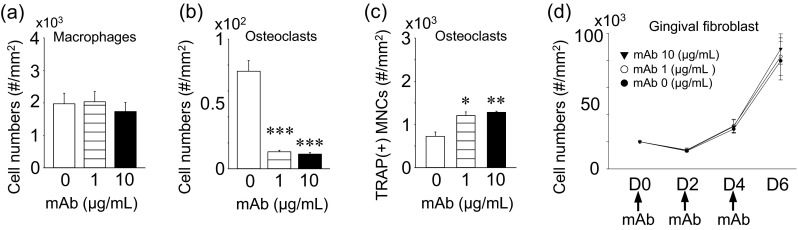
Osteoclast suppression by anti-RANKL mAb in vitro. Primary macrophages were expanded in bone marrow suspension cells by M-CSF treatment. Macrophages were treated with 0, 1, or 10 μg/mL of the anti-RANKL mAb for 3 days and cell numbers (**a**) were counted. Osteoclastogenesis was performed by stimulating macrophages with M-CSF and RANKL. Osteoclast cultures at day 5 were treated with the anti-RANKL mAb for 24 h and stained for TRAP expression. Average osteoclast numbers (**b**) and TRAP(+) macrophages (**c**) were plotted. Primary gingival fibroblasts were isolated from small pieces of gingiva and expanded. Gingival fibroblasts were cultured for 6 days with the mAb treatment every 2 days. Cells were enumerated and plotted (**d**). All assays were triplicated. *p < 0.05, **p < 0.01, ***p < 0.001

**Fig. 3:**
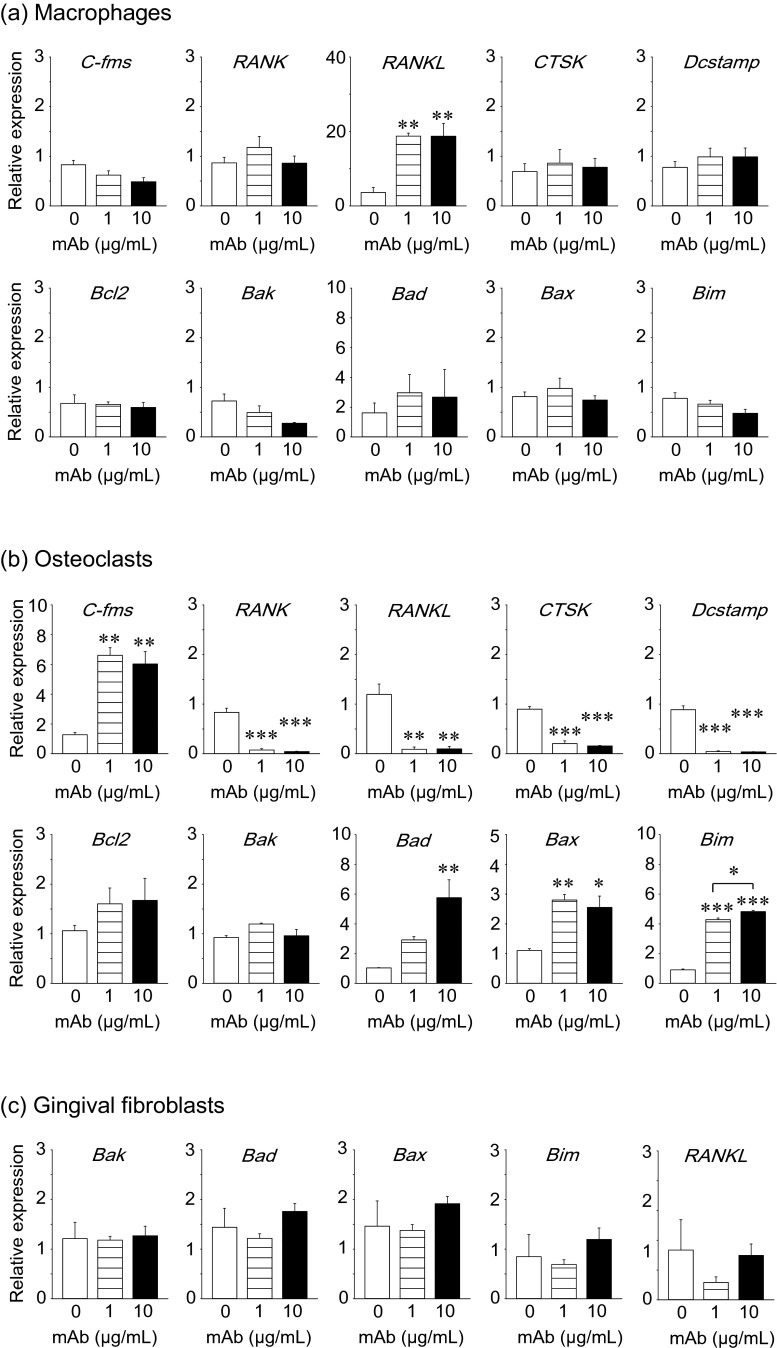
Anti-RANKL mAb treatment upregulated pro-apoptotic genes in osteoclasts in vitro. Primary macrophages were expanded from bone marrow suspension cells by M-CSF treatment. Macrophages were treated with 0, 1, or 10 μg/mL of anti-RANKL mAb for 3 days. Total RNA was extracted, and cDNA was prepared. The expression of osteoclast- and apoptosis-related genes was assessed using qPCR (**a**). Osteoclastogenesis was performed by stimulating macrophages with M-CSF and RANKL. Osteoclast cultures at day 5 were treated with anti-RANKL mAb for 24 h, total RNA was extracted, and cDNA was prepared. The expression of osteoclast- and apoptosis-related genes was assessed using qPCR (**b**). Primary gingival fibroblasts were cultured for 6 days with anti-RANKL mAb treatment every 2 days. At day 6, total RNA was extracted and cDNA was prepared. The expression of *RANKL* and apoptosis-related genes was assessed using qPCR (**c**). All assays were triplicated. *p < 0.05, **p < 0.01, ***p < 0.001

The anti-RANKL mAb treatment showed no effects on macrophage numbers (Fig. 2a), and consistently, no apoptosis-associated genes were regulated by the mAb treatment (Fig. 3a). The expression of *RANKL* was significantly upregulated at the RNA level by the mAb treatment (Fig. 3a). The anti-RANKL mAb treatment significantly suppressed osteoclasts numbers as expected (Fig. 2b). Significantly more TRAP(+) MNCs were observed by the treatment (Fig. 2c). At the RNA level, the expression of osteoclast marker genes including *RANK*, *RANKL*, *CTSK*, and *Dcstamp* were significantly downregulated, but *C-fms* was significantly upregulated by the mAb treatment. The expression of pro-apoptotic genes including *Bad*, *Bax*, and *Bim* were significantly upregulated as expected (Fig. 3b). Primary gingival fibroblasts were treated with the anti-RANKL mAb and enumerated over 6 days. The treatment had no effect on cell numbers (Fig. 2d). The expression of pro-apoptotic genes (*Bak*, *Bad*, *Bax*, and *Bim*) and *RANKL* was not regulated by the mAb treatment in gingival fibroblasts (Fig. 3c).

### Anti-RANKL mAb suppressed osteoclasts but increased TRAP(+) MNCs in vivo

**Fig. 4:**
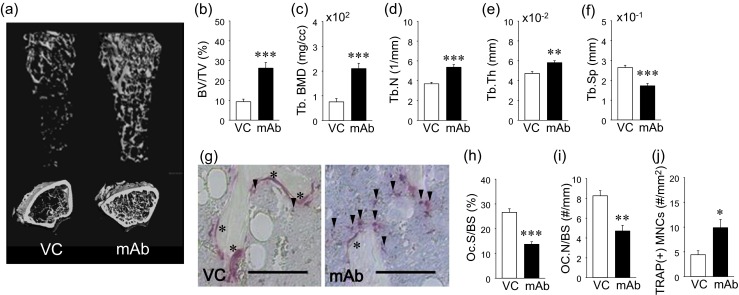
Effect of anti-RANKL mAb in skeleton. Representative reconstructed images of longitudinal and crosssectional femoral microCT data (**a**). Bone volume fraction (*BV/TV*) and trabecular bone mineral density (*Tb.BMD*) were significantly higher in the mAb than VC group (**b** and **c**). Significantly higher trabecular numbers (*Tb.N*) (**d**) and trabecular thickness (*Tb.Th*) (**e**) and significantly lower trabecular separation (*Tb.Sp*) (**f**) were found in the mAb group vs. VC. Representative photomicrographs of TRAP-stained bone sections. *Scale bar* 50 μm (**g**). *Asterisk* indicates osteoclasts on the bone surface and *arrowheads* TRAP(+) MNCs in bone marrow. Osteoclast surface (*OC.S/BS*) (**h**) and osteoclast numbers (*OC.N/BS*) were significantly lower in the mAb group than control (**i**). The numbers of TRAP(+) mononuclear cells (*MNCs*) were significantly higher in the mAb group than control (**j**). *n* ≥ 7/ group; *p < 0.05, **p < 0.01, ***p < 0.001

To confirm that anti-RANKL mAb had the expected antiresorptive effect, the distal femurs were assessed using microCT. The anti-RANKL mAb treatment resulted in a significant increase in bone mass (BV/TV) and trabecular bone mineral density (BMD) compared to VC (Fig. 4a–c). Such an increase in bone mass in the mAb-treated animals was characterized by significantly increased numbers and thickness of trabecular bone and decreased trabecular separation (Fig. 4d–f). Histomorphometric analysis of TRAP-stained sections revealed significantly smaller osteoclast surfaces and reduced osteoclast numbers in the mAb-treated animals vs. VC (Fig. 4g–i), indicating that osteoclast suppression contributed to the increased bone mass observed in the mAb-treated animals. Furthermore, the mAb treatment significantly increased TRAP(+) MNCs in bone marrow compared to VC (Fig. 4j). The TRAP(+) MNCs were predominantly found near but not on the trabecular bone surfaces.

### Altered gene expression in bone marrow by anti-RANKL mAb

**Fig. 5:**
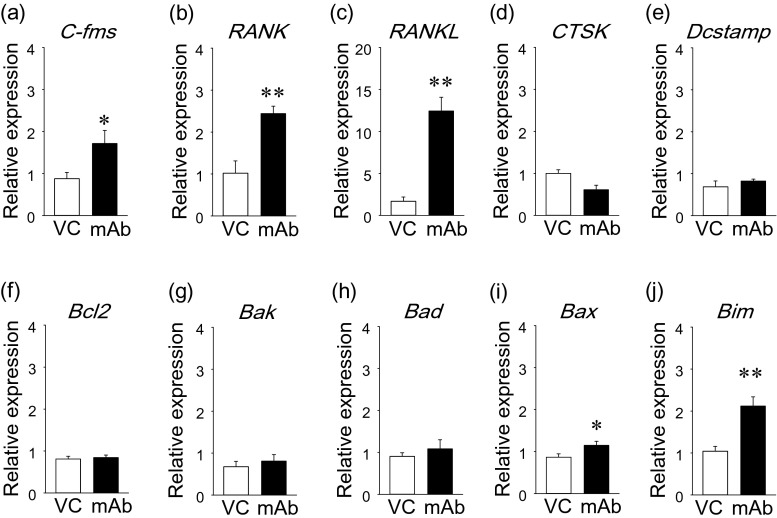
Effect of anti-RANKL mAb on bone marrow gene expression. Gene expression was assessed in bone marrow at the RNA level. Pro-osteoclastic genes (*C-fms*, *RANK*, and *RANKL*), osteoclast genes (*CTSK* and *Dcstamp*), and apoptosis-related genes (*Bcl2*, *Bak*, *Bad* , *Bax*, and *Bim*) were analyzed. Results were normalized by *GAPDH* expression. *C-fms* (**a**), *RANK* (**b**), and *RANKL* (**c**) expression was significantly elevated in the mAb group. No differences were found in the expression of *CTSK* (**d**) and *Dcstamp* (**e**) between the groups. No differences were found in the expression of *Bcl2* (**f**), *Bak* (**g**), and *Bad* (**h**) between the groups. The expression of *Bax* (**i**) and *Bim* (**j**) were significantly stimulated in the mAb group. *n* ≥ 7/ group; *p < 0.05, **p < 0.01

The expression of genes associated with osteoclasts and apoptosis were assessed at the RNA level by qPCR. Anti-RANKL mAb treatment significantly upregulated pro-osteoclast genes including *C-fms*, *RANK*, and *RANKL* in bone marrow (Fig. 5a–c). However, the expression of osteoclast marker genes including *CTSK* and *Dcstamp* were not regulated by the mAb treatment (Fig. 5d, e). The mAb treatment had no effect on the expression of anti-apoptotic gene *Bcl2* and pro-apoptotic genes *Bak* and *Bad* (Fig. 5f–h). However, pro-apoptotic genes, *Bax* and *Bim*, were significantly upregulated by anti-RANKL mAb compared to vehicle control (Fig. 5i, j).

**Fig. 6:**
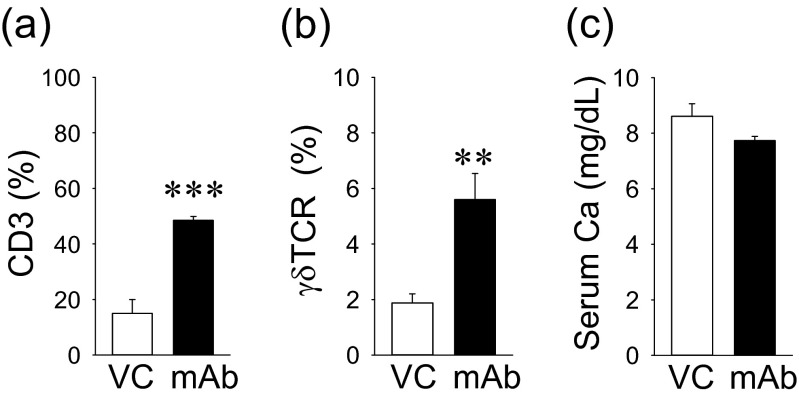
Effect of anti-RANKL mAb on T-cells and serum calcium. The anti-RANKL mAb treatment resulted in a significant increase in CD3(+) T cells (**a**) and γδTCR(+) cells (**b**). No statistical differences were noted in the serum Ca level between the groups (**c**). *n* ≥ 7/ group; **p < 0.01, ***p < 0.001

Since T cells are known to express RANKL, the effect of the anti-RANKL mAb treatment on the T cell population in the bone marrow was assessed. A pan T cell marker, CD3, was used via flow cytometry, and positive cells were significantly increased by the anti-RANKL mAb treatment (Fig. 6a). Additionally, a proportional significant increase in γδTCR(+) cells (a distinct small subset of T cells) was noted in the mAb group vs. VC (Fig. 6b). Despite the osteoclast suppression by anti-RANKL mAb, there were no differences in the serum calcium level between the groups (Fig. 6c).

### Anti-RANKL mAb delays oral wound healing

**Fig. 7:**
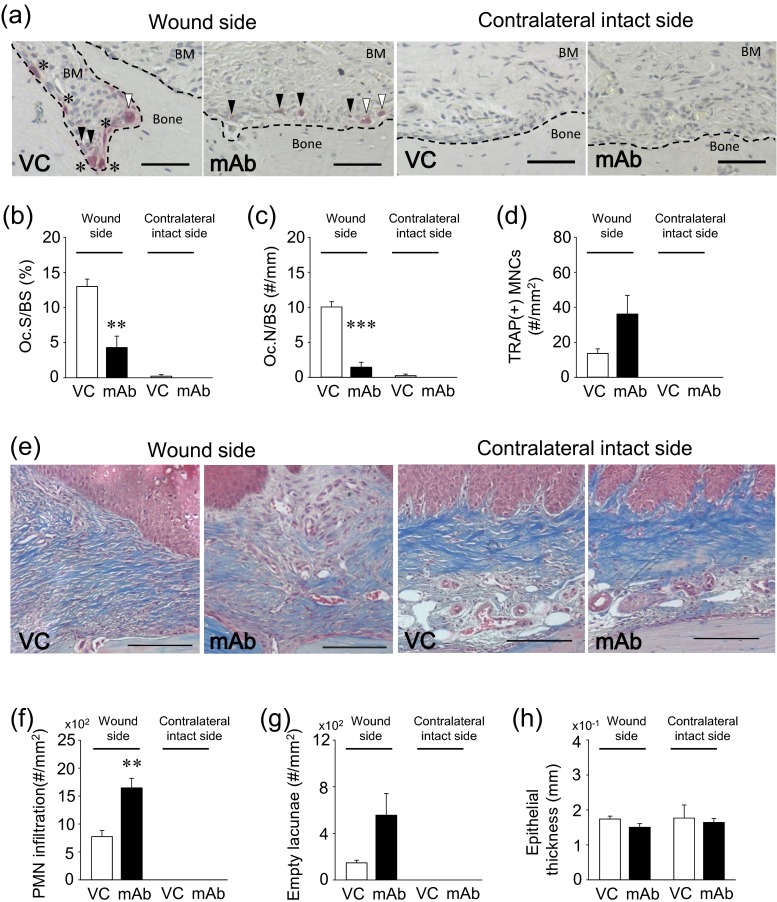
Altered oral osseous wound healing by the anti-RANKL mAb treatment. Representative photomicrographs of TRAP-stained palatal sections. *Asterisk* osteoclasts on the bone surface, *white arrowhead* non-attached osteoclasts, *black arrowhead* TRAP(+) MNCs. TRAP(+) cells were rarely observed in the contralateral intact side. *Scale bar* 50 μm (**a**). Anti-RANKL mAb treatment significantly suppressed osteoclast surface (*OC.S/BS*) and osteoclast number (*OC.N/BS*) compared to VC in the wound side, while osteoclasts were rarely observed regardless of treatment in the contralateral intact side (**a**–**c**). Anti-RANKL mAb treatment increased TRAP(+) MNCs in the palatal connective tissue of the wound side compared to VC treatment (*p* = .058). No TRAP(+) MNCs were detected in the contralateral intact side regardless of treatment (**d**). Representative photomicrographs of Masson’s trichrome-stained palatal sections. *Scale bar* 200 μm (**e**). Significant polymorphonuclear neutrophil (*PMN*) (dark purple nuclei) infiltration was noted in the connective tissue of the wound side in the mAb-treated animals. No PMN infiltration was noted in the contralateral intact side irrespective of treatment (**f**). The numbers of empty osteocyte lacunae were higher in the mAb group than VC (*p* = 0.056) in the wound side. Empty osteocyte lacunae were barely detected in the contralateral intact side regardless of treatment (**g**). No differences were found in the thickness of the healed epithelium (**h**). *n* ≥ 7/ group; **p < 0.01, ***p < 0.001

Histomorphometric analyses were performed to determine whether the anti-RANKL mAb treatment altered oral wound healing after the palate denudation. The assessment of TRAP-stained sections revealed that the anti-RANKL mAb treatment significantly suppressed osteoclasts on the once denuded palatal surfaces compared to VC, while in the contralateral intact side, osteoclasts were rarely observed regardless of the treatment (Fig. 7a). Osteoclast surface (Oc.S/BS) and number (Oc.N/BS) were significantly lower in the mAb group than VC in the wounds, while no effects were noted in the contralateral intact side (Fig. 7b, c). Although not significant (*p* = 0.058), a trend was observed that more TRAP(+) MNCs were observed near the bone surfaces in the mAb group than VC in healing wounds (Fig. 7d). TRAP(+) MNCs were not found in the contralateral intact side. Figure 7e shows the representative photomicrographs of the subepithelial connective tissues in healing wounds. Larger numbers of inflammatory cells (dark purple nuclei) and less collagen fibers (blue) were observed in the healing connective tissue in the mAb group compared to in VC (Fig. 7f). On the other hand, in the contralateral intact side, prominent rete pegs and collagen fibers were observed regardless of treatment. Although a statistical difference was not reached (*p* = 0.056), a clear trend was observed in that larger numbers of empty osteocyte lacunae were noted in the mAb group vs. VC in healing wounds (Fig. 7g). In healing oral wounds, complete epithelium coverage was observed in both groups with no difference in thickness (Fig. 7h).

## Discussion

Since denosumab has no affinity to mouse RANKL, it is a challenge to perform an animal study to understand the denosumab effect in bone. To overcome this difficulty, Kostenuik et al. developed human RANKL knock-in mice, in which denosumab can then be used and have an effect in mice [13]. Although these human RANKL knock-in mice are useful, they exhibit an altered skeletal phenotype from wild-type litter mates. The knock-in mice show increased trabecular bone with decreased osteoclasts [13]. This could be a disadvantage when the denosumab effects are examined in bone as the inherent bone phenotype may affect the outcome. Therefore, the use of an antibody which binds and neutralizes mouse RANKL to a similar degree as denosumab in human is ideal when the goal is to investigate the effect of RANKL inhibition in the mouse skeleton. Furuya et al. reported that a mouse anti-RANKL mAb which was newly developed exhibited similar therapeutic effects to denosumab [14]. A single 5-mg/kg dose of mouse anti-RANKL mAb increased bone mass and BMD in femurs. Since the mouse anti-RANKL mAb treatment is comparable to denosumab treatment in humans, it was used in this study to investigate the effect of the RANKL inhibition in the bone marrow environment and oral osseous wound healing. In this study, we examined the inhibitory effect of mouse anti-RANKL mAb on osteoclasts in vitro. Osteoclasts were induced by M-CSF and RANKL from primary macrophages. When osteoclast cultures were treated with anti-RANKL mAb, the osteoclast number significantly dropped. However, no osteoclastogenesis was disturbed with the vehicle treatment. Since pro-apoptotic *Bad*, *Bax*, and *Bim* were significantly upregulated in the mAb-treated osteoclasts, the significant drop in the osteoclast number was likely due to apoptosis. We further examined whether the anti-RANKL mAb affects macrophages and gingival fibroblasts and found no apoptotic effect on these cell types. Therefore, the mouse anti-RANKL mAb was specific to osteoclasts as anticipated. To gauge its antiresorptive effect in vivo, the anti-RANKL mAb was administered at 5 mg/kg once every 3 weeks for 9 weeks. As expected, the administration of the mAb resulted in significantly high bone mass with suppressed osteoclasts on the bone surfaces, confirming that mouse anti-RANKL mAb exerted sufficient antiresorptive effects in this study via osteoclast suppression.

Significant numbers of TRAP(+) MNCs were noted in the bone marrow of mAb-treated animals. This finding is comparable to our previous finding that long-term bisphosphonate treatment stimulates the development of TRAP(+) MNCs in bone marrow [4]. We thought that this same finding is interesting since the anti-RANKL mAb and bisphosphonates are distinct in their mechanisms of action and pharmacokinetics except that both result in the suppression of osteoclasts. TRAP(+) MNCs are cells in the macrophage/monocyte lineage which are considered immediate precursors of osteoclasts [15]. Therefore, we speculated that increased TRAP(+) MNCs observed in this and previous studies are associated with osteoclast suppression. To get insight into the elevated TRAP(+) MNCs, the expression of pro-osteoclastic, osteoclastic, and apoptotic genes was investigated in bone marrow at the RNA level. It was found that the anti-RANKL mAb stimulated pro-osteoclastic genes including *C-fms*, *RANK*, and *RANKL*, suggesting that the bone marrow environment stimulated myeloid cells to differentiate towards osteoclasts. However, as the anti-RANKL mAb showed no effect on the expression of osteoclast genes including *Dcstamp* and *CTSK*, terminal osteoclast differentiation was not promoted. The expression of *Dcstamp* is required for the fusion of TRAP(+) MNCs [16] and *CTSK* for matrix degradation in osteoclasts [17]. Therefore, in the anti-RANKL mAb-treated animals, the bone marrow environment stimulated osteoclast progenitors to differentiate towards osteoclasts; however, this did not translate into an increase in multinucleated osteoclasts as the mAb treatment inhibited their differentiation and survival. Hence, the result of the gene expression assessment is consistent with the histological finding of high numbers of TRAP(+) MNCs with suppressed osteoclasts on the bone surfaces in the anti-RANKL mAb-treated animals. Osteoclasts are essential cells in bone to maintain physiological skeletal homeostasis. Insufficient osteoclast supply in bone would prompt the marrow environment to stimulate osteoclastogenesis. This is especially true when bone damages accumulate or calcium is in need [18]. When the demand is met, osteoclastogenesis would become inactive, and no strong pro-osteoclastic signals would be generated. However, in the anti-RANKL mAb-treated animals, pro-osteoclastic signals continued, likely due to unsatisfactory osteoclastogenesis. This might result in the accumulation of TRAP(+) MNCs in the bone. Pro-apoptotic *Bax* and *Bim* were upregulated in the anti-RANKL mAb-treated animals. This is likely a reflection of the apoptosis of residing osteoclasts by the anti-RANKL mAb treatment [19, 20]. Since T-cells express abundant RANKL and are therefore important for osteoclastogenesis [21], CD3 and γδTCR(+) cells were assessed. We found significantly high numbers of CD3 and γδTCR(+) cells in the anti-RANKL mAb-treated animals. Interestingly, our previous report also observed elevated γδTCR(+) cells in animals on long-term bisphosphonate treatment [4]. As γδTCR(+) cells play a role in innate immunity [22] and elevated γδTCR(+) cells are implicated in inflammatory diseases [23], osteoclast suppression could marginally influence the immune system.

To determine the effect of the anti-RANKL mAb treatment on oral osseous wound healing, a small area of the palate was denuded, and healing was assessed in histological sections. The contralateral intact side was used as an internal control. No effects of anti-RANKL treatment on TRAP(+) MNCs, the alveolar bone, and the epithelial layer were observed in the contralateral intact side. Consistently, the results of the in vitro assays showed that the enumeration of gingival fibroblasts and macrophages, which reflects a sum of proliferation and apoptosis, was not affected by the mAb treatment. The mAb treatment did not cause inflammation in the connective tissue when no wounds were created. On the other hand, in the wound side, significant PMN infiltration was noted in the connective tissue of the mAb-treated animals. Furthermore, considerably reduced osteoclasts with an increased number of TRAP(+) MNCs were found in the mAb-treated animals. Thus, inflammation with suppressed osteoclasts was particularly associated with the wounds in the anti-RANKL mAb-treated animals. These observations are quite comparable to our previous observation where the bisphosphonate zoledronic acid was administered to inhibit osteoclasts [4, 24]. Therefore, regardless of whether bisphosphonates or anti-RANKL mAb are used, osteoclast suppression delays osseous healing and causes the retention of inflammation in the soft tissue. The sustained inflammation could be sterile inflammation caused by damage-associated molecular pattern molecules from necrotic osteocytes [25] or pathogen-associated inflammation caused by locally infected bone. Although the sustained inflammation was local, it may have a systemic impact. It is possible that the elevated γδTCR(+) cells in the bone marrow were associated with the sustained inflammation in the oral wounds.

In summary, this study clearly shows that mouse anti-RANKL mAb treatment increased bone mass by suppressing osteoclasts. However, the anti-RANKL mAb treatment resulted in increased TRAP(+) MNCs in bone marrow and altered oral osseous wound healing. Osteoclastic repair of the damaged palate was delayed and inflammation sustained due to osteoclast suppression. Although the current study used the mouse anti-RANKL mAb, findings were comparable to those of a study where zoledronic acid was used. Therefore, increased TRAP(+) MNCs are associated with osteoclast suppression, not specifically bisphosphonates or anti-RANKL mAb.
